# Optimization of Ternary In_x_Ga_1-x_N Quantum Wells on GaN Microdisks for Full-Color GaN Micro-LEDs

**DOI:** 10.3390/nano13131922

**Published:** 2023-06-23

**Authors:** Yu-Chung Lin, Ikai Lo, Cheng-Da Tsai, Ying-Chieh Wang, Hui-Chun Huang, Chu-An Li, Mitch M. C. Chou, Ting-Chang Chang

**Affiliations:** 1 Department of Physics, National Sun Yat-sen University, Kaohsiung 80424, Taiwan; d092030001@nsysu.edu.tw (Y.-C.L.); d002030010@student.nsysu.edu.tw (C.-D.T.); tcchang3708@gmail.com (T.-C.C.); 2 Center for Nanoscience and Nanotechnology, National Sun Yat-sen University, Kaohsiung 80424, Taiwan; d982030006@nsysu.edu.tw; 3 Department of Materials and Optoelectronic Science, National Sun Yat-sen University, Kaohsiung 80424, Taiwan; m9036601@student.nsysu.edu.tw (H.-C.H.); chuanli@mail.nsysu.edu.tw (C.-A.L.); mitch@faculty.nsysu.edu.tw (M.M.C.C.)

**Keywords:** full-color GaN micro-LEDs, In_x_Ga_1-x_N-based quantum wells (QW), plasma-assisted molecular beam epitaxy (PAMBE)

## Abstract

Red, green, and blue light In_x_Ga_1−x_N multiple quantum wells have been grown on GaN/γ-LiAlO_2_ microdisk substrates by plasma-assisted molecular beam epitaxy. We established a mechanism to optimize the self-assembly growth with ball-stick model for In_x_Ga_1-x_N multiple quantum well microdisks by bottom-up nanotechnology. We showed that three different red, green, and blue lighting micro-LEDs can be made of one single material (In_x_Ga_1-x_N) solely by tuning the indium content. We also demonstrated that one can fabricate a beautiful In_x_Ga_1-x_N-QW microdisk by choosing an appropriate buffer layer for optoelectronic applications.

## 1. Introduction

Group-III nitride compounds have been extensively studied in the past decades because of their applications in optoelectronic [1,2], high-power electronic [3,4], and spintronic devices [5,6]. In recent years, GaN-based micro-light-emitting diodes (micro-LEDs) have attracted more attention to their application in small-size illuminating displays, such as smart watches, virtual reality, augmented reality, etc. There are two methods to fabricate the GaN micro-LEDs: one is to reduce the epifilm size by a photo-lithography etching process (so-called top-down nanotechnology), and the other is direct self-assembly of GaN microdisk (named as bottom-up nanotechnology), which was established and developed by our group earlier [7,8,9]. The band structure of ternary group-III nitride compounds can be engineered from binary compounds (i.e., AlN, GaN, and InN) with Vegard’s equation [10]: ***E***_g_(In_x_Ga_1−x_N) = ***E***_g_(GaN)*(1-x) + ***E***_g_(InN)*x − b*x*(1-x), where the bandgap of AlN (***E***_g_ = 6.15 eV), GaN (***E***_g_ = 3.43 eV), and InN(***E***_g_ = 0.64 eV) [10,11]. The bowing factors were then established for the bandgaps of ternary compounds for AlGaN, InGaN, and InAlN [12,13,14,15]. However, the epitaxial growth of ternary compounds has to face the obstacle of lattice mismatching among AlN, GaN, and InN. An appropriate substrate (such as sapphire [1,2], SiC [16], ZnO [17], Si(111) [18], LiAlO_2_ [19], and LiGaO_2_ [20], etc.) is then chosen for different purposes to grow the epifilms of ternary compounds with the help of buffer layers to reduce the effect of a lattice mismatch. However, when one applies the top-down nanotechnology to fabricate nanodevices (e.g., micro-LED), the edge damage of the nanodevice sidewall by the direct photo-lithography etching process always degrades the performance of nanodevices [21]. Therefore, Lo et al. developed a low-temperature growth mechanism with a ball-stick model of bottom-up nanotechnology for self-assembly epi-growth of hexagonal ***c***-plane wurtzite GaN microdisk on γ-LiAlO_2_ (LAO) substrate to avoid the edge-damage by using plasma-assisted molecular beam epitaxy (PAMBE) [7,12,22,23]. A hexagonal basis ***c***-plane [000$\bar{1}$] growth of GaN and a rectangular basis ***M***-plane [1$\bar{1}$00] growth of GaN can be provided at the same time [7]. From our previous study, we found that the Si (111) substrate can only produce 3D ***c***-plane GaN [0001] nanorods instead of 3D ***c***-plane GaN [000$\bar{1}$] microdisks due to the property of the wurtzite GaN lattice structure [18], and the GaN microdisk can be used as a nearly free-standing substrate for the growth of ternary InGaN/GaN quantum wells (QW) for further optoelectronic applications [22]. On the other hand, according to the growth mechanism for a 28° angle GaN hexagonal microdisk, we can control the size of the microdisk by the duration time of epifilm growth for the three-dimensional (3D) self-assembling GaN microdisk thereafter. A finite growth of 3D hexagonal ***c***-plane [000$\bar{1}$] GaN can be then grown atop the LAO substrate [8], and the nearly free-standing GaN/LAO substrate offers an ideal platform to study the ternary In_x_Ga_1-x_N quantum wells for the application of full-color micro-LEDs.

By using the GaN microdisk as a nearly free-standing substrate to grow the ternary In_x_Ga_1-x_N/GaN quantum wells, we can engineer the ternary In_x_Ga_1-x_N/GaN micro-LED quantum wells by tuning the indium composition (*x*) to cover the full spectrum of visible light under the size limit as small as micrometers for the application of micro-LED illuminating displays. However, the strain induced by the lattice mismatch among AlN, GaN, and InN still needs to be resolved when we use the GaN microdisk as a nearly free-standing substrate to engineer the ternary In_x_Ga_1-x_N/GaN quantum wells. In our previous study, we found that an anisotropic strain was built in a wurtzite GaN microdisk as grown on the ***c***-plane GaN/LAO microdisk substrate [24]. The anisotropic strain will create a crack on the heterostructured epifilms during the epitaxial growth of ternary compounds (e.g., InGaN and InAlN), when the strain in the heterostructures exceeds an elastic limit of wurtzite lattice (i.e., at the indium composition difference, Δ*x*~13%) [25]. The size control of the GaN microdisk allowed us to grow a finite 3D self-assembling ternary In_x_Ga_1-x_N/GaN quantum well, before the crack was formed (i.e., under the elastic strain limit). For instance, a buffer layer (e.g., Δ*x* < 13%) can be inserted between the GaN and In_0.13_Ga_0.87_N layer to grow a hexagonal In_0.13_Ga_0.87_N/GaN quantum well microdisk without any creation of cracks for a blue light micro-LED. Two-step buffer layers can be used to grow the ternary In_x_Ga_1-x_N quantum wells with further indium contents (e.g., *x* > 25%) for green and red light micro-LEDs, and so on. In this paper, we employ a two-stepped InGaN buffer layer to grow the ternary In_x_Ga_1-x_N quantum wells with *x* > 0.25 on the microdisk GaN/LAO substrate at different growth temperatures, and a temperature-dependent mechanism of indium-incorporation is established to optimize the InGaN/GaN microdisk quantum wells for the application of red-green-blue micro-LED displays.

## 2. Materials and Methods

**Figure 1:**
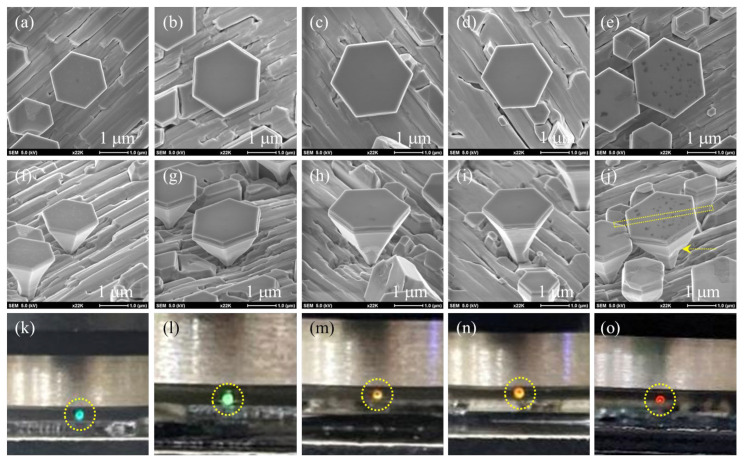
(**a**), (**b**), (**c**), (**d**) and (**e**) are top views of SEM images with a scale bar of 1 μm of Samples A–E, respectively. (**f**), (**g**), (**h**), (**i**) and (**j**) are the tilted views of SEM images with a scale bar of 1 μm of Samples A–E, respectively. (**k**), (**l**), (**m**), (**n**) and(**o**) are directly visible image by the He-Cd 325 nm laser focus on the microdisks from Samples A–E, respectively.

The In_x_Ga_1-x_N/In_y_Ga_1-y_N multiple quantum wells (MQWs) atop GaN microdisks were grown on high quality 1 × 1 cm^2^ γ-LiAlO_2_ (LAO) substrates by plasma-assisted molecular beam epitaxy (Veeco Applied-GEN 930 system) with Ga- and In-evaporation from standard effusion cells and N_2_-plasma sources with 450 W from the rf-plasma cell. The LAO substrates were cleaned by the following step in sequence before mounting on the holder of the MBE: (i) acetone, isopropanol, and phosphoric acid (1:30) for 5 min of each; (ii) de-ionized water for 5 s; (iii) dried by nitrogen gas. After completing the cleaning step above, the LAO substrates were outgassed in molecular beam epitaxy (MBE) chamber at 770 °C for 10 min. Upon completing the above procedure, the temperature of substrate was decreased down to the specified growth temperatures for the study. In our previous studies, more detail of the epi-growth can be referred [7,8,9]. The wetting layer, Ga, was first deposited for 5 min at 630 °C on the LAO substrate, and then the two-step method (i.e., the N/Ga flux ratio 29.0 performed for 35 min at first, then increased to 138.8 performed for 70 min) was used to fabricate the GaN microdisks as a strain-free substrate (e.g., GaN/LAO nearly free-standing substrate) for the further growth of In_x_Ga_1-x_N/In_y_Ga_1-y_N MQWs. The evaluation of flux ratio was calculated by the beam equivalent pressure (BEP) of evaporative sources from effusion cell versus the N_2_ source from the rf-plasma cell. To grow InGaN quantum wells that achieved red light emission, the stepped InGaN buffer layers had been employed for the epi-growth and a series of growth-temperature dependence on In_x_Ga_1-x_N MQWs had been studied. The sample structures and different growth parameters for each sample are listed in Table 1. For Sample A, the stepped InGaN buffer layers were grown at a fixed N flux ratio (BEP = 9.0 × 10^−6^ torr) with different In/Ga ratio to be 0.5 (i.e., In flux ratio BEP = 5.0 × 10^−8^ torr and Ga flux ratio BEP = 1.0 × 10^−7^ torr) and 1.67 (i.e., In flux ratio BEP = 1.0 × 10^−7^ torr and Ga flux ratio BEP = 6.0 × 10^−8^ torr) for each layer (InGaN buffer layers 1 and 2). The InGaN buffer layer 1 was grown by 20 min at 720 °C with lower indium (In) concentration, which was estimated to be lattice-matched with strained GaN microdisks (e.g., under the elastic limit). When InGaN buffer layer 1 was firmly established, the InGaN buffer layer 2 with higher In concentration (*x*) was then applied to tune the lattice constant of ternary In_x_Ga_1-x_N quantum well toward a red light-emitting micro-LED structure. We designed Sample A as grown with the two InGaN buffer layers only to verify the quality and In concentration (*x*) of the buffer layers for comparison basis. For Samples B–E, the identical two-step buffer layers with Sample A were used, and In_x_Ga_1-x_N quantum wells with the In/Ga flux ratio to be 7.5 (i.e., In flux ratio BEP = 1.5 × 10^−7^ torr and Ga flux ratio BEP = 2.0 × 10^−8^ torr) were grown right after InGaN buffer layer 2 for 3 min by different temperatures from 720 °C to 670 °C. The In_x_Ga_1-x_N quantum wells were intervened by the In_y_Ga_1-y_N barrier, which was grown at 720 °C for 3 min (the same parameter as InGaN buffer layer 2) to form an In_x_Ga_1-x_N/In_y_Ga_1-y_N quantum well, and repeated with five periods (i.e., 5-period MQW). Finally, an In_y_Ga_1-y_N cap was grown for 5 min to terminate the MQW structure in Samples B–E. To simplify the crystal structure, five In_x_Ga_1-x_N/In_y_Ga_1-y_N multiple quantum wells on GaN microdisks with a diameter of ~2.5 μm were selected from Samples A–E for the study of a red light-emitting micro-LED structure. Figure 1a–j show the scanning electron microscope (SEM) images of top view and tilted view for the samples with the scale bar of 1 μm taken by the electron beam of the dual-beam focus ion beam (Hitachi NX2000 system). The optical properties of the microdisks were analyzed by photoluminescence (PL) measurement with a He-Cd 325 nm laser (HORIBA HR800 system). In addition, the red light microdisk (Sample E) was selected to double-check the optical properties by cathodoluminescence (CL) measurement (JEOL JSM-6330 system), and the micro-structure of Sample E with the zone axis of [11$\bar{2}$0] was characterized by the high-resolution transmission electron microscope (HR-TEM) measurement (Tecnai F20G2 MAT S-TWIN system). The dual-beam focus ion beam system (Hitachi NX2000 system) was used to prepare the HR-TEM specimen.

## 3. Results and Discussion

**Figure 2:**
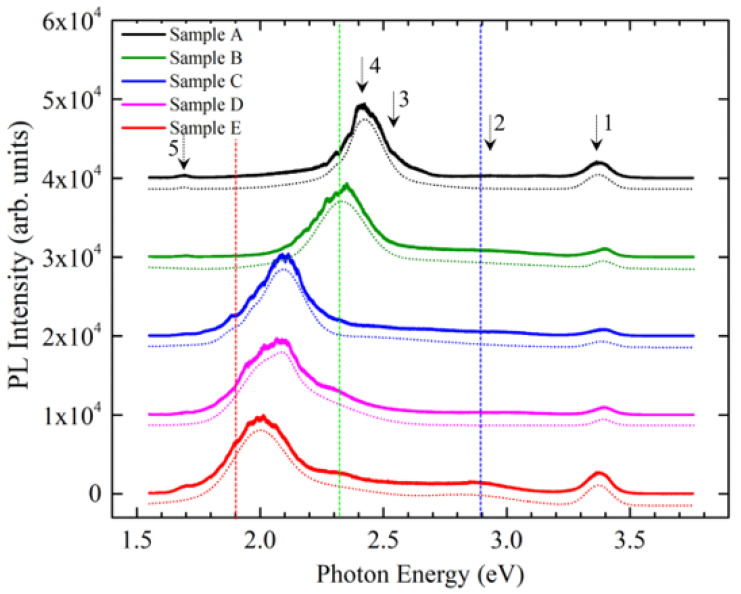
The normalized PL spectra at room temperature for Samples A–E. The *z*-axis had been offset for comparison and the dot line show the non-linear cure fitting for Samples A–E, respectively.

**Figure 3:**
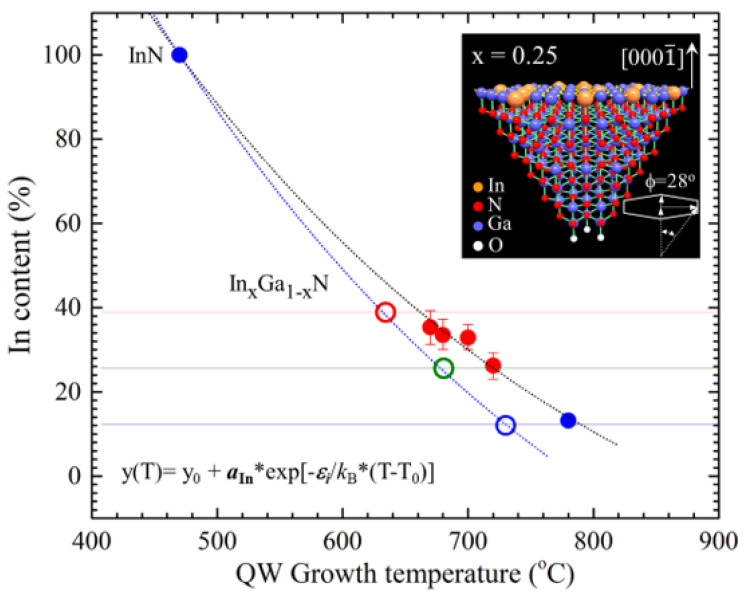
The plot of indium content versus QW growth temperature with the non-linear curve fitting exponential function.

To analyze the optical properties of the In_x_Ga_1-x_N/In_y_Ga_1-y_N MQW microdisks, we performed photoluminescence (PL) measurements on the selected microdisks from Samples A–E. Figure 2 shows the result of normalized PL measurements for Samples A–E at room temperature with a He-Cd 325 nm laser, the CCD of the detector with the resolution of 1024 ×512 pixel, and the 40× objective lens for UV Lasers. The optical images of these microdisks were taken during the PL measurements as shown with highlighted dotted circles in Figure 1k–o. The images show the visible lights emitted from these microdisks (eye-witnessed). The full spectrums of PL measurements were plotted in Figure 2 for the five samples; the red-, green-, and blue-dotted lines roughly indicated the photon energies of red, green, and blue lights. We used a non-linear curve fitting method with five Gaussian functions to fit these PL spectrums, and the best fitting results were shown by the dotted curves below. Two major peaks (Peaks 1 and 4) were detected. It was found that Peak 1 at (3.370 ± 0.0001) eV, with a full width half maximum (FWHM) of (0.1107 ± 0.0001) eV, did not shift with different samples, which was attributed to the light emitted from GaN layer. We also noted that Peak 4 at (2.422 ± 0.001) eV, with FWHM = (0.1852 ± 0.0002) eV, for Sample A exhibited one order of amplitude greater than Peak 1, and the photon energy was about the green light shown in Figure 1k. Therefore, we contributed the light of major peak (4) to the In_x_Ga_1-x_N buffer layers for Sample A, which did not include the 5-period In_x_Ga_1-x_N/In_y_Ga1-yN MQWs. Thus, indium composition (*x*) of the In_x_Ga_1-x_N buffer layers for Sample A, corresponding to the photon energy (2.422 ± 0.001) eV, can be evaluated from Vegard’s equation: ***E***_g_(*x*) = [3.43 − 2.79 × (*x*) − 1.9 × (1 − *x*) × *x*] eV [10], and the indium composition is equal to 23.8%. However, after the insertion of 5-period In_x_Ga_1-x_N/In_y_Ga1-yN MQWs, the major peak (4) shifted to a lower energy for Sample B–Sample E. Figure 1l–o show the directly visible images by the He-Cd 325 nm laser focus on the microdisks for Sample B–Sample E, respectively. We observed that the green light of Sample A gradually shifted to red light, with the photon energies of (2.332 ± 0.001) eV, (2.092 ± 0.001) eV, (2.072 ± 0.001) eV, and (1.995 ± 0.001) eV, respectively, and the corresponding FWHMs of (0.2207 ± 0.0002) eV, (0.2148 ± 0.0003) eV, (0.2313 ± 0.0005) eV, and (0.2568 ± 0.0006) eV. It indicated that the quality of the MQWs for Sample B–Sample E was consistent with the FWHM within 0.2568 eV when the indium content of MQWs changed. The other peaks (e.g., Peaks 2 and 3) will be attributed to the impurities or structural defects (e.g., dislocations, stacked faults, etc.), and Peak 5 was the secondary diffraction of the emission from the GaN (Peak 1). Again, we evaluated the indium composition of MQWs for Sample B–Sample E by Vegard’s equation and obtained the content of indium in In_y_Ga_1-y_N quantum wells to be 26.2%, 32.9%, 33.5%, and 35.3% for Samples B, C, D, and E, respectively. We plotted the indium content against the QWs growth temperature in Figure 3 for the four samples (the red solid cycles). With the inset point as shown by blue solid circles, the content of the indium in the InN microdisk (i.e., 100%) [23] and in the In_0.13_Ga_0.87_N microdisk disk at 13% [8] referred to our previous papers, and we plotted with the best fitting exponential function: y(T) = y_0_ + *a*_In_ * exp [−*ε_i_*/*k*_B_ × (T − T_0_)], where y_0_ is an constant from the background, *a*_In_ is the possibility of indium incorporation, *k*_B_ is Boltzmann constant, T is QW growth temperature, and *ε_i_* is a coefficient related to indium incorporation (as shown with the black dotted curve in Figure 3). It can be found that the indium incorporation would be increasing as the temperature decreases.

**Figure 4:**
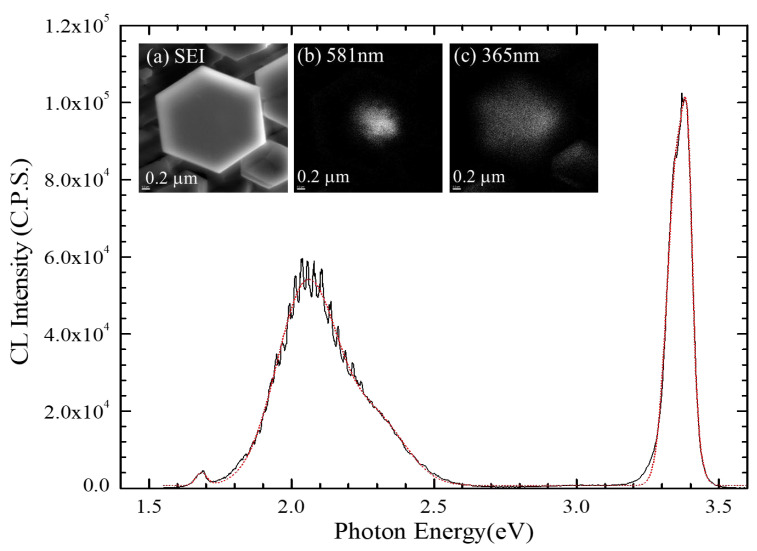
The CL spectrum measured at room temperature for In_y_Ga_1-y_N/In_x_Ga_1-x_N MQWs of Sample E, and the dot line showed the non-linear curve fitting. The insets show the SEI (**a**) and CL images (**b**) and, (**c**) at room temperature for wavelength of 581 nm and 365 nm, respectively.

**Figure 5:**
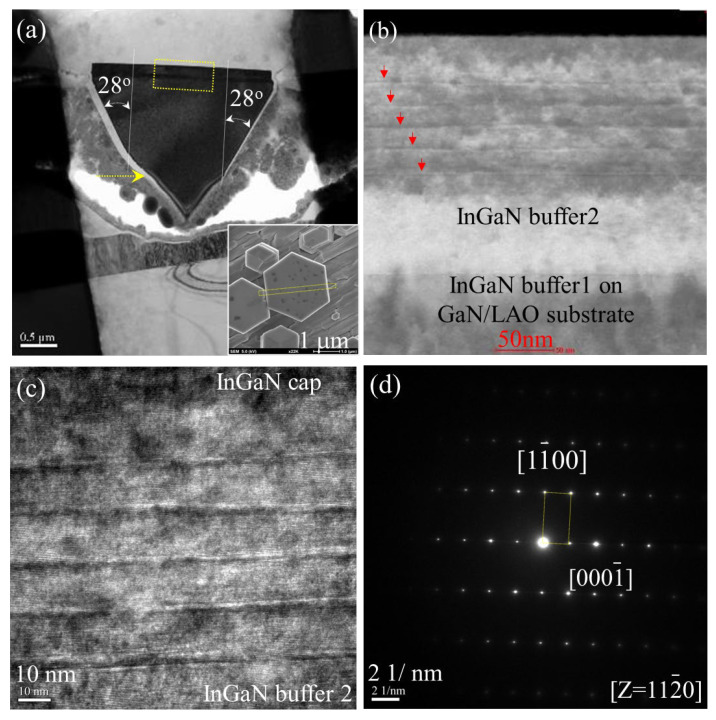
(**a**) TEM image for the In_y_Ga_1-y_N/In_x_Ga_1-x_N MQWs microdisk with a scale bar of 0.5 μm. The inset showed SEM top-view images with cleavage plane along [1$\bar{1}$00] direction (scale bar is 1 μm). (**b**) The STEM image taken at the top area of GaN microdisk with scale bar of 50 nm. (**c**) The high-resolution TEM image of In_y_Ga_1-y_N/In_x_Ga_1-x_N MQWs on the top of the microdisk with the scale bar of 10 nm. (**d**) The SAD pattern taken at the top area with the scale bar of 2 (1/nm).

In order to examine the microstructure of MQWs and its optical properties, the In_x_Ga_1-x_N/In_y_Ga_1-y_N microdisk of Sample E, which was the one used for PL measurement in Figure 1e, was selected for the evaluation of the light-emitting source by the measurements of cathodoluminescence (CL) and secondary electron images (SEI). The result of CL measurement at room temperature is shown in Figure 4, and the images are shown in the insets of Figure 4a–c. The CL spectrum was obtained by the following setting: (i) the range of detecting photon energy at 1.77 eV to 3.75 eV, with accelerative voltage of 10 kV, and (ii) 1400 V for the extraction voltage of photoelectric magnitude tube. The CL spectrum was analyzed by fitting non-linear Gaussian functions, and we obtained two major peaks at (2.056 ± 0.001) eV with FWHM equal to 0.2536 eV and (3.390 ± 0.0002) eV with FWHM equal to 0.037 eV. As compared with the CL images at the wavelength of 581 nm (equivalent to 2.134 eV) as shown in Figure 4b, it was attributed to the emission of In_y_Ga_1-y_N layer of the microdisk at the center of Figure 4a. Many branches occurred at the major peak of (2.056 ± 0.001) eV and possibly contributed to the interferometric fringes owing to the thickness of the epitaxial stacking of multiple quantum wells in In_x_Ga_1-x_N/In_y_Ga_1-y_N microdisk. On the other hand, for the CL image Figure 4c at wavelength of 365 nm (equivalent to 3.397 eV), the peak was attributed to the wurtzite GaN layer of the microdisk located at the center of Figure 4a, and the CL image also was shown at the right-hand side in Figure 4c, which was attributed to the smaller microdisk as shown in the right-hand side of the central microdisk in SEI picture, Figure 4a. The two major peaks of CL spectrum are consistent with the PL results (i.e., Sample E in Figure 2). In order to investigate the microstructure of the MQWs microdisk in more detail, the same microdisk (i.e., Sample E) shown in Figure 1e was used for the high-resolution TEM measurement. As shown in the insert of Figure 5a, the cleavage direction [1$\bar{1}$00] of microdisk TEM specimen was prepared. From the TEM image in Figure 5a, we observed that the multiple QWs (the yellow rectangular) were grown atop the GaN microdisk on the LAO substrate. It was found that the 28° angle awl-shaped GaN microdisk was established before introducing indium atoms. After introducing the indium atoms, the 28° angle cannot be held at the first In_x_Ga_1-x_N layer due to the strain and a vertical growth along the ***c***-axis, which occurred for the first In_x_Ga_1-x_N buffer layer. In addition, it was the reason for terminating the 28°-angle divergent awl-shaped growth. We noted that the buffer layers repaired the structural defects to keep 28° angle growth as pointed out by the yellow arrow in Figure 5a, which was obviously exhibited in the SEM tilted image in Figure 1j. The scanning transmission electron microscopy (STEM) measurement was performed on the multiple QWs (the yellow rectangular) to show the high contract images for the In_x_Ga_1-x_N/In_y_Ga_1-y_N MQWs grown between GaN disk and InGaN cap. The STEM result is shown in Figure 5b. It clearly exhibits the microstructure of the whole In_x_Ga_1-x_N/In_y_Ga_1-y_N MQWs GaN microdisk. We can observe that the In_x_Ga_1-x_N/In_y_Ga_1-y_N MQWs were well-formed after the growth of InGaN buffer layer 2. As indicated by the arrow in the Figure 5b, it clearly exhibits five layers of In_x_Ga_1-x_N/In_y_Ga_1-y_N MQWs, as we designed. The crystalline micro-structure for the In_x_Ga_1-x_N/In_y_Ga_1-y_N MQWs was reconfirmed by the high-resolution TEM images as shown in Figure 5c. We can clearly observe the micro-fluctuated In_y_Ga_1-y_N epi-layer was sandwiched by two well-established In_x_Ga_1-x_N layers to form an In_x_Ga_1-x_N/In_y_Ga_1-y_N/In_x_Ga_1-x_N QW and repeated for five times to form the whole In_y_Ga_1-y_N/In_x_Ga_1-x_N MQWs. Clearly, it shows that it was similar as the STEM image shown in Figure 5b. The thickness of wells (In_y_Ga_1-y_N) and barriers (In_x_Ga_1-x_N) are about 2.8 nm and 10.7 nm, yielding the growth rate to be 0.93 nm/min for the In_y_Ga_1-y_N wells and 3.56 nm/min for the In_x_Ga_1-x_N barrier. The selected area diffraction (SAD) measurement was performed on the In_y_Ga_1-y_N/In_x_Ga_1-x_N QWs layer. From the SAD pattern in Figure 5d, we evaluated the ***d***-spacing of the In_y_Ga_1-y_N was 0.5350 nm. As a result, the indium composition of In_y_Ga_1-y_N layer can be calculated by a linear approximation of wurtzite lattice constant: y = [***d_c_***(In_y_Ga_y_N)-***d_c_***(GaN)]/[***d_c_***(InN)-***d_c_***(GaN)] = 36.6%, where we used ***d_c_***(InN) = 0.5703 nm and ***d_c_***(GaN) = 0.5146 nm. This result shows good agreement with PL analysis (i.e., 35.3%).

**Figure 6:**
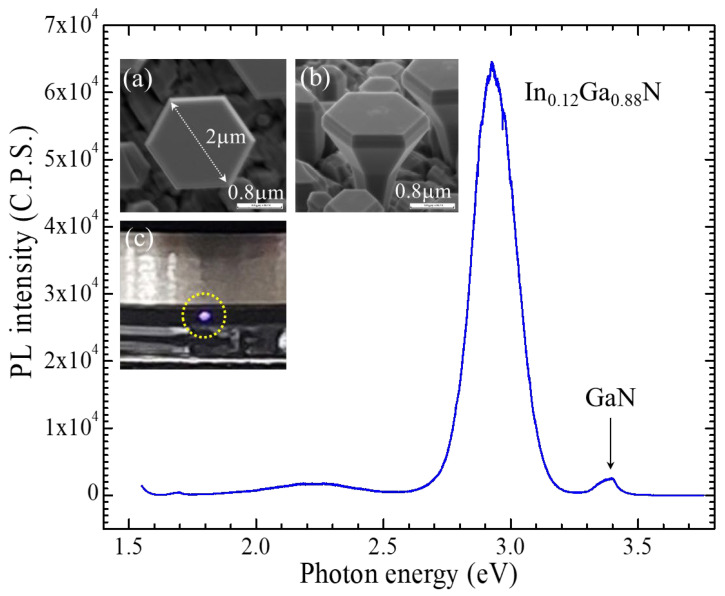
The PL spectrum of blue In_x_Ga_1-x_N quantum-well microdisk with a simpler structure. The insets show (**a**) the top-view, (**b**) tilted-view SEM images, and (**c**) optical image of the sample.

Molecular beam epitaxy (MBE) was performed in an ultra-high vacuum environment, and the thermodynamic mechanisms of absorption, desorption, diffusion, and coalescence interplayed with adatoms to achieve high-quality epifilms during the epitaxial growth. By tuning the evaporation temperature of element sources (e.g., In, Ga, and N), we can control the momentum of elements impinging on the substrate surface, and then the temperature of the substrate (i.e., growth temperature) will smoothly govern the thermodynamic mechanisms to form the epi-layers. Applying the plasma-assisted MBE to the “bottom-up nanotechnology” to grow 3D wurtzite ***c***-plane GaN microdisks [7,8,9], the absorption and desorption play more important roles on the small size surface (e.g., <2 μm). Because of the specific lattice structure of wurtzite, the cation atoms (i.e., In and Ga) and anion atoms (i.e., N) deposited layer-by-layer (cation-layer/anion-layer) on the ***c***-plane surface of GaN [000$\bar{1}$] for self-assembling microdisks, as shown in the inset of Figure 3. Therefore, the indium incorporation on the cation layer will simply compete with gallium incorporation atop the nitrogen anion layer. The momentums (*p* = *mv*) of evaporative indium and gallium can be controlled by the temperature of effusion cells. The gallium atoms becomes more active than indium atoms due to smaller mass (*m*) and smaller atomic size when impinging on the nitrogen anion layer with the same substrate temperature (i.e., growth temperature). This is the reason why high growth temperature will be favorable to the gallium cation layer growth. In Figure 3, the plot of indium incorporation (%) versus growth temperature, derived from the experiments in this study, showed a thermodynamic exponential equation (black dotted curve): y(T) = y_0_ + *a*_In_ * exp [−*ε_i_*/*k*_B_ * (T − T_0_)]. Thus, one can optimize the growth of ternary In_x_Ga_1-x_N quantum wells by changing the epitaxy growth background (e.g., y_0_ and T_0_) and plot the phenomenological exponential equation by fixing the factors (i.e., *a*_In_ and *ε_i_*) systematically to evaluate the best growth conditions. For instance, we grew another blue In_x_Ga_1-x_N quantum well microdisk with a simpler structure as shown in Figure 6. The growth parameters for the sequence were: (i) GaN microdisk buffer on LAO substrate with N-flux(9 × 10^−6^ torr)/Ga-flux(1.25 × 10^−7^ torr), 40 min at 630 °C; (ii) Ga/N layer with N-flux(9 × 10^−6^ torr)/N-flux(6.50 × 10^−8^ torr), 100 min at 630 °C; (iii) InGaN triple QW with N-flux(9 × 10^−9^ torr)/Ga-flux(5.40 × 10^−8^ torr)/In-flux(5.40 × 10^−8^ torr), 90 s at 730 °C; and finally (iv) GaN cap with N-flux(9 × 10^−6^ torr)/Ga-flux(1.25 × 10^−7^ torr), 3 min at 770 °C. The beautiful shaped microdisk is shown in the insets of Figure 6a,b. The PL spectrum of the sample showed the major peak at (2.935 ± 0.001) eV with FWHM of (0.1959 ± 0.0001) eV, which is one order of amplitude greater than that of GaN. The indium composition derived from Vegard’s equation is 12%. We replotted the exponential equation with the sample parameter (i.e., 730 °C, 12%) and approached InN by fixing the factors (i.e., *a*_In_ and *ε_i_*), as shown in the blue dotted curve in Figure 3. In such a way, one can systematically evaluate the growth parameters similar to the blue microdisk from the exponential equation. For example, one can design green or red In_x_Ga_1-x_N QW microdisks, e.g., green at (680 °C, 26%) or red at (630 °C, 39%) for the RGB micro-LED applications.

## 4. Conclusions

We have grown red, green, and blue light In_x_Ga_1-x_N multiple quantum wells on GaN/LAO microdisk substrates by PAMBE. From the PL measurements, we found that the major peak shifted to red as the MQWs growth temperature decreased from 720 °C to 670 °C. From the diagram of indium incorporation rate versus QW growth temperature, we established a mechanism to optimize In_x_Ga_1-x_N multiple quantum wells microdisks for micro-LEDs. We showed that, for the first time, three different red, green, and blue lighting micro-LEDs can be made of one single material (In_x_Ga_1-x_N) by self-assembling nanotechnology. We also demonstrated that one can fabricate a beautiful In_x_Ga_1-x_N-QW microdisk by choosing an appropriate buffer layer for optoelectronic applications.

## Figures and Tables

**Table 1:** Sample structure and growth parameters for the In/Ga flux ratio and substrate temperature.

	Sample A	Sample B	Sample C	Sample D	Sample E
Sample structure	Metal flux ratio/Growth Temperature (°C)				
InGaN buffer 1	0.5/720	0.5/720	0.5/720	0.5/720	0.5/720
InGaN buffer 2	1.67/720	1.67/720	1.67/720	1.67/720	1.67/720
InGaN QWs (**x5**)		**7.5/720**	**7.5/700**	**7.5/680**	**7.5/670**
InGaN barriers		1.67/720	1.67/720	1.67/720	1.67/720
InGaN cap		1.67/720	1.67/720	1.67/720	1.67/720

## Data Availability

The data presented in this study are available on request from the corresponding author.
